# Nanocomposites of NiO/CuO Based MOF with rGO: An Efficient and Robust Electrocatalyst for Methanol Oxidation Reaction in DMFC

**DOI:** 10.3390/nano10081601

**Published:** 2020-08-15

**Authors:** Tayyaba Noor, Sadaf Pervaiz, Naseem Iqbal, Habib Nasir, Neelam Zaman, Muhammad Sharif, Erum Pervaiz

**Affiliations:** 1 School of Chemical & Materials Engineering (SCME), National University of Sciences and Technology (NUST), H-12 Campus, Islamabad 44000, Pakistan; erum.pervaiz@scme.nust.edu.pk; 2 School of Natural Sciences (SNS), National University of Sciences and Technology (NUST), H-12 Campus, Islamabad 44000, Pakistan; spervaiz.mschem17sns@student.nust.edu.pk (S.P.); habibnasir@sns.nust.edu.pk (H.N.); 3 U.S-Pakistan Center for Advanced Studies in Energy (USPCAS-E), National University of Sciences and Technology (NUST), H-12 Campus, Islamabad 44000, Pakistan; naseem@uspcase.nust.edu.pk (N.I.); nzaman.ese19ces@student.nust.edu.pk (N.Z.); 4 Department of Chemistry, King Fahd University of Petroleum and Minerals, Dhahran 31261, Saudi Arabia; msharif@kfupm.edu.sa

**Keywords:** metal–organic framework (MOF), reduced graphene oxide (rGO), methanol oxidation reaction (MOR), cyclic voltammetry (CV)

## Abstract

In this work a novel bimetallic nickel oxide/copper oxide metal–organic framework (NiO/CuO MOF) has been developed by using two linkers: Benzene Dicarboxylic acid (BDC) and Pyrazine. The composites of NiO/CuO MOF with different amounts of reduced graphene oxide (rGO) were synthesized through a hydrothermal method and subsequently characterized by multiple significant techniques like XRD, SEM, EDX, FTIR and Raman IR for an investigation of their structural and morphological properties. The prepared series of material was later employed for electrochemical oxidation of methanol, tested by cyclic voltammetry (CV) in basic medium on a modified glassy carbon electrode (GCE). The electrochemical response depicts that increasing concentration of rGO enhances the electrocatalytic activity of the catalyst for methanol oxidation reaction (MOR). The catalyzed oxidation reaction of methanol by NiO/CuO MOF and rGO-NiO/CuO MOF composites give a superlative current density of 437. 28 mA/cm^2^ at 0.9 V potential at 50 mV/s scan rate. This activity makes it a promising catalytic material for electrolysis of methanol in direct methanol fuel cell (DMFC).

## 1. Introduction

Fuel cells are promising candidates to provide sustainable and efficient energy supply for advanced portable electrical vehicles and electronic devices [1]. A catalysis reaction occurs between fuel (methanol, ethanol and molecular hydrogen) at anode and molecular oxygen at cathode, which converts chemical energy into electrical energy via electrochemical processes [2]. These cells have proved ideal energy conversion devices and assured appropriate electrical supply for the generation of power. Although the initial development of fuel cells was based on large scale application with the ultimate emphasis on the development of high energy cells (200–300 kW), now intense development of smaller fuel cells (50–75 kW) is dominating due to the edge of their low emission characteristics [3]. Though fuel cells put forward the most clean possible power production with much lower emissions of greenhouse gases and are efficient in energy conversion of fuel into power in contrast to thermal power plants and gasoline engines, the commercial potential of fuel cells depends on its ability to decrease the cost and use of expensive materials like catalysts [4]. The incentive is to shift away from fossil fuels and hydrocarbons to solve the environmental pollution-related issues. The establishment of a never ending source of energy, mitigating environmental pollution and consumption of fossil fuels, to support an increasingly flourishing world are among the most influential issues in recent years [5]. The rapidly increasing energy crisis of the world means we are in search of alternate energy sources due to the rapid development of portable electric devices and electronic vehicles [6]. DMFCs have gained numerous attentions by its convenient fuel transportation, low environmental pollution, high energy conversion, suitable supply and storage [7]. According to the survey, the fuel cell marketplace for power generation projects and contracts funded with ~$100 million in 2003 [8].

Numerous electro catalysts have been applied to oxidize methanol but the highest electro catalytic activity is achieved by Pt-based alloys. Being an inert metal, platinum offers definite advantages in the applications of fuel cells, like compliance in acidic electrolytes [9,10]. Although Pt and Pt-based catalysts are common and efficient for catalysis, there are still some significant obstacles plaguing in the use of such catalysts like high cost, limited availability, poor stability and susceptibility towards methanol crossover [11]. Moreover, the kinetics of methanol oxidation slow down the adsorption of carbon monoxide on Pt surface; when Pt interacts with oxygen containing carbon species (mainly CO) produced in methanol, oxidation is so strong that active sites of Pt are covered and blocked which lead to recession of activity [12,13,14]. Yet these obstacles have been reduced by non-precious and more economical metal catalysts like nickel-, iron-, cobalt- and copper-based compounds and their composites with graphene oxide (GO), reduced graphene oxide (rGO), and carbon nano tubes etc. [15] also reported as a major competitor to those composites which are based on Pt nanoparticles [16]. Catalytic oxidation of methanol produces hydrogen ion and CO_2_ which can be represented as [17]

CH_3_OH + H_2_O → CO_2_ + 6H^+^ + 6e^−^ E_o_ = 0.02 V (1)

The methanol oxidation reaction over metal catalysts is both practically and significantly attractive. These reactions may produce a vast variety of products, like methyl formate, dimethyl ether, formaldehyde, dimethoxymethane, and formic acid. The selectivity depends on conditions of the reaction such as, partial pressure of the reactants, conversion and reaction temperature [18]. 

Copper provides significant electro catalytic activity when it comes to oxidation but by adding a small amount of good alloying materials to copper has been reported to give a dramatic increase in its electro catalytic activity as compared to pure copper electrode material. For instance, adding 5% of Mn to Cu, represented as Mn_5_Cu_95_, shows a significant improvement in glucose oxidation occurring electrochemically in alkaline media [19]. Nickel acquires significant attention as a catalyst due to its surface oxidation properties i.e., inter conversion of Ni^2+^ and Ni^3+^ [20]. Many nickel-involving materials have been served as catalysts in various fuel cells, and nickel has been widely used as an electro catalyst for both cathodic as well as anodic reactions in electrolysis of water and organic synthesis [21]. Van Effen and Evans reported ethanol oxidation in KOH solution that results in the production of nickel oxide with higher valency that serves as an oxidizing agent. The fact was supported by impedance spectroscopy and cyclic voltammetry [22]. There is a vast variety of compositions of Ni/Cu alloys due to the fact that, in pure form, both metals (i.e., Nickel, Copper) have the same face centered cubic (fcc) arrangement with the same lattice parameters (α = 3.523 for Ni and α = 3.616 for Cu). Khulbe et al. presented an excellent review on the behavior of Ni/Cu alloys in various catalytic mechanisms such as conversion of ortho-para hydrogen, H_2_/D_2_ exchange reaction and some hydrogenation reactions [23].

Carbon-supported materials have high surface and area are widely used to catalyze electrochemical oxidation reaction of methanol [24]. Graphene-based compounds are appreciated in this regard due to their efficient conductance, low cost, large surface area and high mechanical strength. Furthermore, graphene-based catalysts increase electron and mass transport of reactants [25]. In comparison to reduced graphene oxide (rGO), graphene oxide (GO) shows less efficiency so reduction process is required to obtain better properties with the aid of a reducing agent for the elimination of functional groups of oxygen [26].

Metal–organic frameworks (MOFs) illustrate remarkable electro catalytic activities for oxidation of alcohols [27]. MOFs have some unique properties that make them appreciable catalysts for the previously mentioned reaction. Firstly, they acquire surface areas in the range of 500–6500 m^2^ g^−1^ and pore sizes of about 3–35 Å [28]. Secondly, the surface properties of MOFs can be altered by versatile organic ligands that bear different functional groups on the surface channel. Sites for adsorption of gas can be introduced in MOFs which gives selectivity for adsorbents and a versatile range of storage capacities [29]. The major purpose of this work is to analyze the electrochemical oxidation reaction of methanol on novel NiO/CuO bimetallic MOF and its composites with 1, 2, 3, 4, 5 and 8 wt% rGO in a solution of 3M methanol and 1M NaOH.

## 2. Experimental

### 2.1. Materials

Nickel nitrate hexahydrate (Ni(NO_3_)_2_·6H_2_O), copper nitrate trihydrate (Cu(NO_3_)_2_·3H_2_O), pyrazine (C_4_H_4_N_2_), 1, 4-benzenedicarboxylic acid (BDC), N, N-dimethylformamide (DMF), sodium nitrate (NaNO_3_), graphite powder, conc. sulfuric acid (H_2_SO_4_), hydrogen peroxide (H_2_O_2_), potassium permanganate (KMnO_4_), hydrazine hydrate (NH_2_NH_2_·H_2_O), sodium hydroxide (NaOH), nafion (C_7_HF_13_O_5_S·C_2_F_4_), methanol (CH_3_OH) and DI water used in this work were purchased from Sigma-Aldrich (St. Louis, MO, USA) and Merck((Kenilworth, NJ, USA)) of analytical grade and were used without further purification.

### 2.2. Synthesis of Bimetallic Nickel Oxide/Copper Oxide Metal–Organic Framework (NiO/CuO MOF)

NiO/CuO MOF was synthesized by using hydrothermal method [30,31]. Pyrazine (0.32 g) and terephthalic acid (0.33 g) was dissolved in 14 mL of DMF with continuous stirring. After that, equimolar of Cu(NO_3_)_2_·3H_2_O and Ni(NO_3_)_2_·6H_2_O (1 mmol) was added to the solution with continuous stirring until all solid was dissolved to form a homogenous solution. This solution was poured into Teflon-lined autoclave and heated for 48 h at 200 °C in heating oven. Bluish green colored product was obtained washed with ethanol for the removal of unreacted substances and collected after drying at 55 °C under vacuum. Thermal stability of NiO/CuO MOF under synthesis condition is determined by TGA (supporting material Figure S1).

### 2.3. Synthesis of Graphene Oxide (GO)

Graphene Oxide was synthesized by reported Hummers’ method [32]. Powdered graphite flakes (4 g) and 4 g of sodium nitrate (NaNO_3_) were added to 100 mL of sulfuric acid (H_2_SO_4_) with continuous stirring for 2 h. Afterwards, 12 g of potassium permanganate (KMnO_4_) was added to the suspension pinch by pinch while temperatures were brought to 35 ± 3 °C with continuous stirring for another 48 h for complete oxidation. Water (200 mL) was then added slowly, maintaining the temperature below 60 °C and 400 mL of water was added for further dilution followed by addition of 10 mL hydrogen peroxide and stirring for 30 min [32]. The color of solution turned yellowish brown. The reaction mixture was centrifuged and washed repeatedly with dilute HCl and DI water to make certain that the pH was about 6–7 and product was dried under vacuum for 24 h at 60 °C [33]. The resultant graphene oxide was in the form of fine sheets.

### 2.4. Synthesis of Reduced Graphene Oxide (rGO)

For the synthesis of rGO, 100 mg GO was dissolved in 100 mL of DI water *followed by sonication for 2 h to form a homogenous aqueous suspension of brown color.* Hydrazine hydrate (1 mL) was added to the suspension and refluxed for 24 h at 100 °C. A *black flocculent kind of substance appeared on the surface of the reaction mixture gradually.* The product was filtered, washed and dried under vacuum at 45 °C [34].

### 2.5. Synthesis of rGO-NiO/CuO MOF Composites

NiO/CuO MOF and its composites with rGO i.e., (1–5 wt%, 8 wt%) were synthesized by the reported hydrothermal method [6,30]. A solution with 0.33 g of terephthalic acid and 0.32 g of pyrazine was prepared in 14 mL of DMF followed by the addition of equimolar salts of Cu(NO_3_)_2_·3H_2_O and Ni(NO_3_)_2_·6H_2_O with continuous stirring until all solids were dissolved to make a clear solution. Then rGO (1–5, 8 wt%) was added to the solution and stirring was continued for another hour. Afterwards the solution was transferred to Teflon-lined autoclave and heated to 200 °C for 48 h. Product was collected, washed with ethanol and dried under vacuum at 55 °C.

## 3. Material Characterization

Perkin Elmer’s Spectrum 100 (PerkinElmer, Waltham, MA, USA) was used for FTIR analysis to predict the presence of functional groups and Thermo Scientific DXR 2 Smart Raman (Thermo Fischer Scientific, Waltham, MA, United States) (3500–500 cm^−1^) for Raman analysis of graphene oxide and reduced graphene oxide. Crystalline structure of prepared catalyst was examined by powder X-ray diffractometer (STOE, Darmstadt, Germany) having Cu Ka at λ = 1.540608 Å at 2θ range from 5° to 80°. Surface morphology and shape were characterized by Scanning electron microscopy (VEGA3 TESCAN, Brno, Czech Republic).

## 4. Electrochemical Measurements of the Electrocatalysts

Electrochemical measurements were performed by using three techniques (cyclic voltammetry (CV), *electrochemical impedance spectroscopy* (EIS) and Chronoamperometry) in 1M NaOH + 3M CH_3_OH through a conventional three-electrode system supported by Gamry instrument with Reference 3000/3000AE which was equipped with data acquisition software version 7.06. In this system, ink of NiO/CuO MOF catalyst was deposited on GC surface of electrode by micro pipette which serves as working electrode, Ag/AgCl was served as a reference electrode and Pt wire acted as counter electrode. In this study, methanol was consumed as fuel, NaOH acted as support to electrolytes, 5 wt% Nafion served as binding agent that provided sufficient conductivity [35] and ethanol was used as solvent.

### Preparation of Working Electrode

For the evaluation of electrochemical performance of synthesized MOFs by cyclic voltammetry (CV), electrochemical impedance spectroscopy (EIS), Chronoamperometry (CA) measurements, catalytic ink was prepared by mixing catalyst (2 mg), ethanol (100 µL) and 5 wt% solution of Nafion (20 µL) [36]. The resulting suspension was coated onto a glassy carbon electrode (GCE) with the help of a micro pipette. Drying of modified electrode was carried out at room temperature before every electrochemical measurement.

## 5. Results and Discussion

NiO/CuO MOF and all of its composites with rGO i.e., 1 wt% rGO–NiO/CuO MOF, 2 wt% rGO–NiO/CuO MOF, 3 wt% rGO–NiO/CuO MOF, 4 wt% rGO–NiO/CuO MOF, 5 wt% rGO–NiO/CuO MOF and 8 wt% rGO–NiO/CuO MOF were characterized through FTIR, PXRD, SEM and EDX techniques.

**Figure 1:**
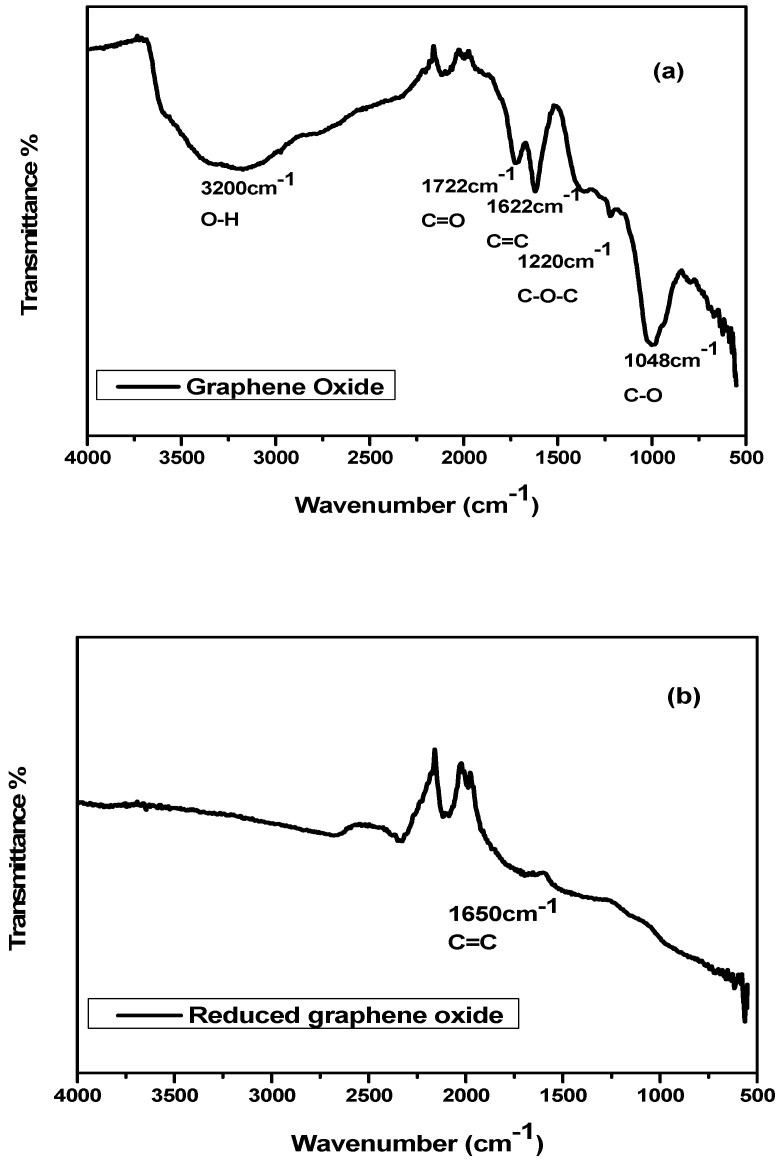

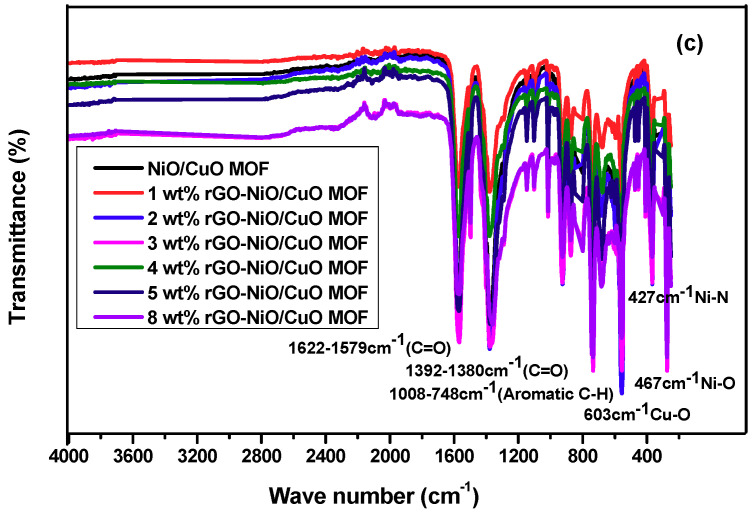
FTIR spectra of (**a**) graphene oxide (GO) (**b**) reduced graphene oxide (rGO) (**c**) nickel oxide/copper oxide metal–organic framework (NiO/CuO MOF) and its composites with 1 wt%, 2 wt%, 3 wt%, 4 wt% and 5 wt% rGO–NiO/CuO MOF.

Figure 1a shows FTIR spectrum of graphene oxide along with absorption peaks of stretching vibrations at 3200 cm^–1^ for O–H, 1722 cm^−1^ for C=O, 1622 cm^–1^ for C=C bond. Peaks at 1220 cm^–1^ and 1048 cm^−1^ are due to stretching vibrations of epoxy and alkoxy (C–O and C–O–C) groups. The existence of these functional groups support the successful synthesis of graphene oxide (GO) from graphite [37].

Figure 1b illustrated FT-IR spectra of reduced graphene oxide (rGO). Peaks at 1722 cm^−1^ and 1220 cm^−1^ are absent in rGO which support the removal of oxygen groups from sheets of GO on reduction. Furthermore, the peak at 1650 cm^−1^ compliments the recovery of sp^2^ lattice in graphene [38].

The presence of functional groups in NiO/CuO MOF and its composites with rGO were also investigated through FTIR. In Figure 1c, the sharp peak at 1622–1579 cm^−1^ and 1392–1380 cm^−1^ indicate the C=O asymmetric and symmetric stretching in all prepared catalysts. The absence of a strong absorption peak at around 1715–1680 cm^−1^ confirms the deprotonation of –COOH group in benzene dicarboxylic acid. Furthermore, the peak at 1008–748 cm^−1^ indicates the presence of aromatic C–H in both in plane and out of plane bending vibrations in benzene dicarboxylic acid linkers and peaks at 603 cm^−1^, 441–550 cm^−1^, 427 cm^−1^, 467 cm^−1^ illustrate Cu–O, Cu–N, Ni–N and Ni–O stretching vibrations, respectively [39].

**Figure 2:**
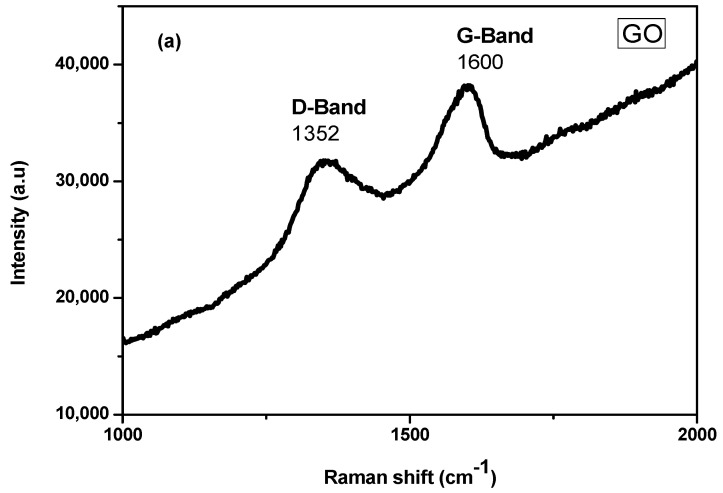

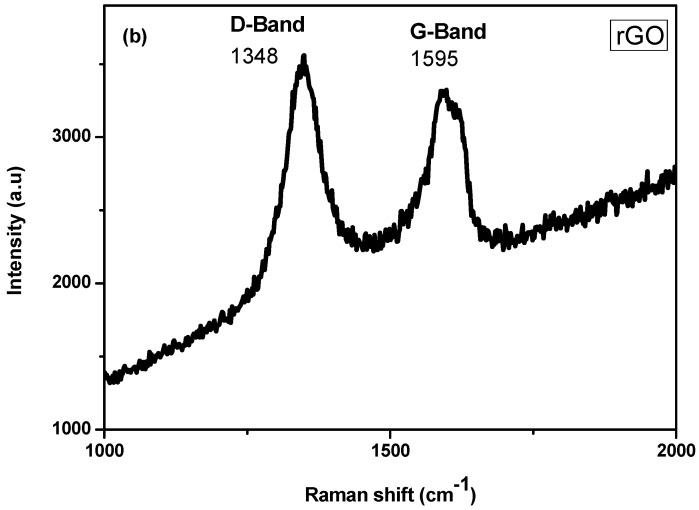
Raman Spectra of (**a**) graphene oxide (GO) (**b**) reduced graphene oxide (rGO).

Figure 2a represents the Raman spectrum of GO. Structural defects present in graphene layers can be investigated by Raman spectra. Two characteristic bands can be observed in graphene oxide spectrum i.e., D band at 1352 cm^−1^ and G band at 1600 cm^−1^. G band appears due to C=C stretching vibrations of the aromatic ring while D band appears due to sp^2^ carbon disorder in the aromatic ring system, formation of sp^3^ carbon in C=C and the functional groups which contain oxygen present on the graphene oxide layer. Intensity ratios (I_D_/I_G_) of D and G band displayed structural disorder and defects. This ratio is almost 0.92 in the structure of GO which supports the oxidation of graphite.

Furthermore, reduced graphene oxide Figure 2b also shows two bands. Because of the defects present in rGO, D band appears at 1348 cm^−1^, while G band of rGO displayed at about 1595 cm^−1^. This G band appears due to stretching vibration of sp^2^ hybridized carbon present in C=C in the reduced graphene layer. The value of intensity ratios (I_D_/I_G_) is about 1.42 in the case of the reduced graphene oxide layer which is complicated by the insufficient recovery of graphene structure and presence of sp^3^ defects in the structure [40,41].

**Figure 3:**
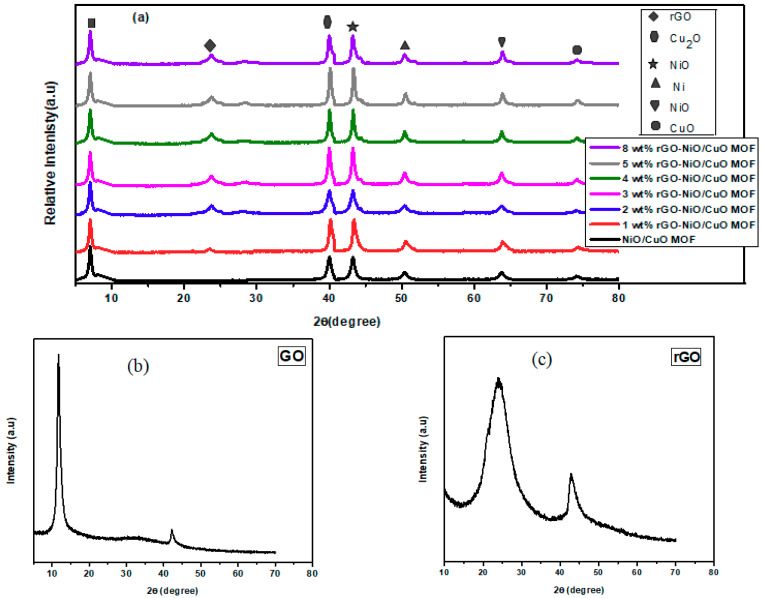
Powder XRD of (**a**) NiO/CuO MOF, 1 wt% rGO–NiO/CuO MOF, 2 wt% rGO–NiO/CuO MOF, 3 wt% rGO–NiO/CuO MOF, 4 wt% rGO–NiO/CuO MOF, 5 wt% rGO–NiO/CuO MOF and 8 wt% rGO–NiO/CuO MOF (**b**) graphene oxide (**c**) reduced graphene oxide.

The XRD analysis of prepared NiO/CuO MOF and its composites with rGO is shown in Figure 3a. All the diffraction peaks can be indexed to crystalline NiO/CuO MOF and rGO-NiO/CuO MOF composites. In the XRD pattern, existence of sharp peak at position (220) at 2θ value of 8.7° reflects the crystallinity of the MOF [42]. Furthermore, a broad peak at around 27° corresponds to rGO suggesting the graphene sheet ordering along their stacking direction and characteristic peak at around 43.25° correspond to nickel oxide (NiO JCPDS card No. # 04-0835) [43,44,45]. The other characteristic peaks at positions (202) and (311) at 2θ value of 50.44° and 74.1°, respectively, correspond to copper oxide (CuO JCPDS card No. # 48-1548) [46].

Figure 3b demonstrate the XRD pattern of GO (graphene oxide). Graphene oxide formation was proved by angle 2θ of 10.5° with 0.841 nm d spacing. A less intense peak at 2θ of 42.2° with d spacing of 0.212 nm shows the presence of unexfoliated graphite.

Figure 3c demonstrates the XRD pattern of rGO (reduced graphene oxide). Reduced graphene oxide formation was confirmed by an angle 2θ of 25.4° with 0.4 nm d spacing with a broad peak that corresponds to the reduced graphene oxide. A less intense peak at 2θ of 42.5° with d spacing of 0.21 nm corresponds to unexfoliated graphite presence [47].

**Figure 4:**
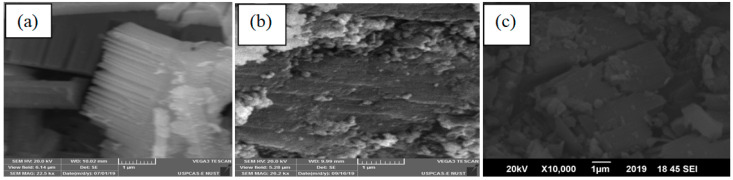

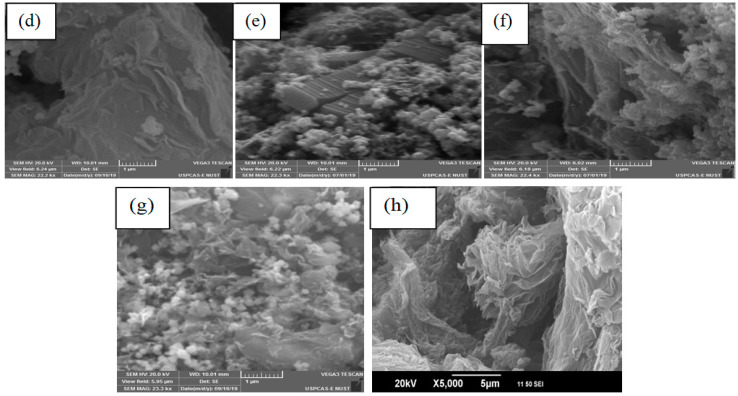
(**a**) NiO/CuO MOF, (**b**) 1 wt% rGO–NiO/CuO MOF, (**c**) 2 wt% rGO–NiO/CuO MOF, (**d**) 3 wt% rGO–NiO/CuO MOF, (**e**) 4 wt% rGO–NiO/CuO MOF, (**f**) 5 wt% rGO–NiO/CuO MOF (**g**) 8 wt% rGO–NiO/CuO MOF and (**h**) rGO.

All the synthesized catalysts’ morphologies were also studied through SEM (scanning electron microscopy) analysis illustrated in Figure 4. SEM images show clear incorporation of NiO/CuO MOF nanosheets stacked over each other within the wrinkled sheets of rGO [48,49]. While EDX results in Table 1 depicts the presence of nickel and copper along with carbon and oxygen. The gradual increase in carbon content reveals that composites were successfully synthesized without any impurity.

**Figure 5:**
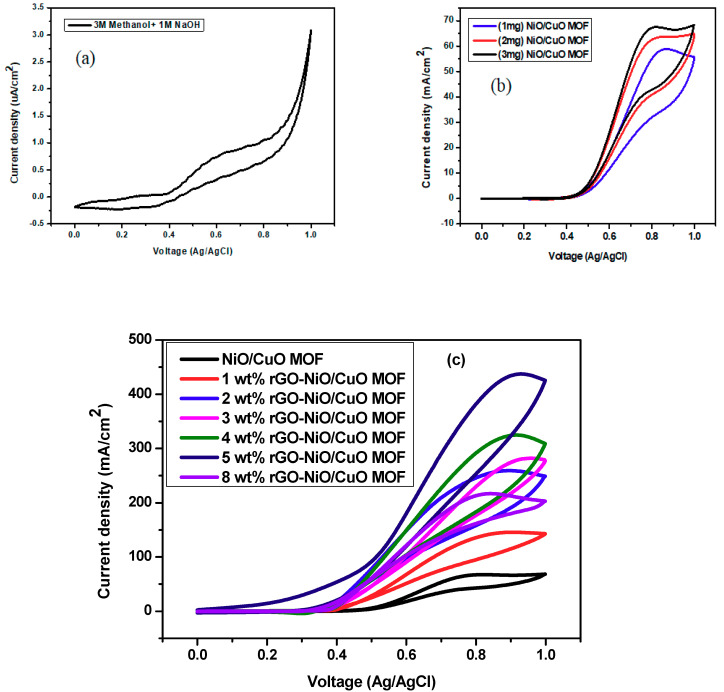
Cyclic Voltamograms of (**a**) bare glassy carbon electrode (GCE) (**b**) NiO/CuO MOF in different concentrations (**c**) composites with 1 wt%, 2 wt%, 3 wt%, 4 wt%, 5 wt% and 8 wt% rGO/NiO/CuO MOF in 3M CH_3_OH and 1M NaOH at 50 mV/s.

Electrochemical activity of NiO/CuO MOF and rGO–NiO/CuO MOF composites was tested by cyclic voltammetry in 3 M CH_3_OH and 1 M NaOH at a scan rate of 50 mV/s for methanol oxidation reaction. Current density of bare electrode was 0.81 µA/cm^2^ as shown in Figure 5a. Concentration studies for NiO/CuO MOF were carried out at various catalyst loadings such as 1.0 mg, 2.0 mg and 3.0 mg as illustrated in Figure 5b, which clearly shows the gradual increase in current density with more active sites available for methanol oxidation reaction ultimately enhancing the catalytic activity [50]. Gradual increase in peak current density with increased concentration of rGO in comparison to pure NiO/CuO MOF can be shown in Figure 5c. This means that rGO shows active performance towards methanol oxidation reaction [51]. This characteristic is important for its electrochemical application in fuel cells [50]. The improved electro catalytic activity of NiO/CuO MOF with rGO composite is due to their synergic effect. Presence of rGO increased the surface area of the catalyst and also supported their charge transfer properties [42]. Pure NiO/CuO MOF shows current density of 67.48 mA/cm^2^ at scan rate 50 mV/s. NiO/CuO MOF with 5 wt% rGO shows a maximum current density at 50 mV/s of 437.28 mA/cm^2^ at 0.9 V. With the gradual increase in amount of rGO, peak current density rises to the following levels: 145.5 mA/cm^2^ for 1 wt%, 259 mA/cm^2^ for 2 wt%, 264.57 mA/cm^2^ for 3 wt%, and 324 mA/cm^2^ for 4 wt% at a scan rate of 50 mV/s. The catalyst with 5 wt% rGO shows maximum response as compared to other 1–4, 8 wt% and NiO/CuO MOF as shown in Table 2. This increased response of 5 wt% rGO composites clearly shows that it lowers the activation energy required for methanol oxidation reaction.

rGO–NiO/CuO MOF (8 wt%) was also tested for the optimization purpose of reduced graphene oxide in a methanol oxidation reaction as shown in Figure 5c. In this case, obtained results were out of trend as it shows lower current density than 5 wt% rGO–NiO/CuO MOF. Most probably this is due to the fact that the large amount of rGO results in incomplete dispersion at the time of synthesis which led to covering of the surface of the MOF which blocked the pores and catalytic sites of the catalyst and lowered its performance [34,52]. So, it is suggested that current density can be enhanced by increasing rGO to a certain limit; although, increasing quantity of rGO may have a negative effect on current density.

**Figure 6:**
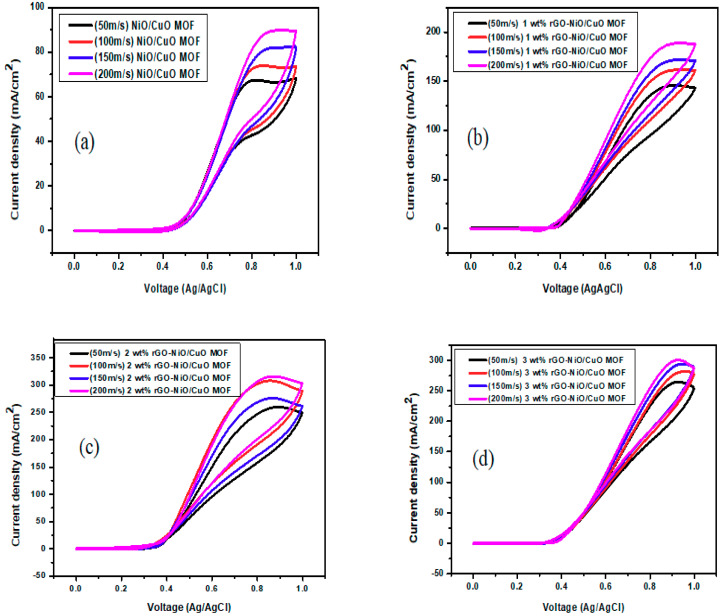

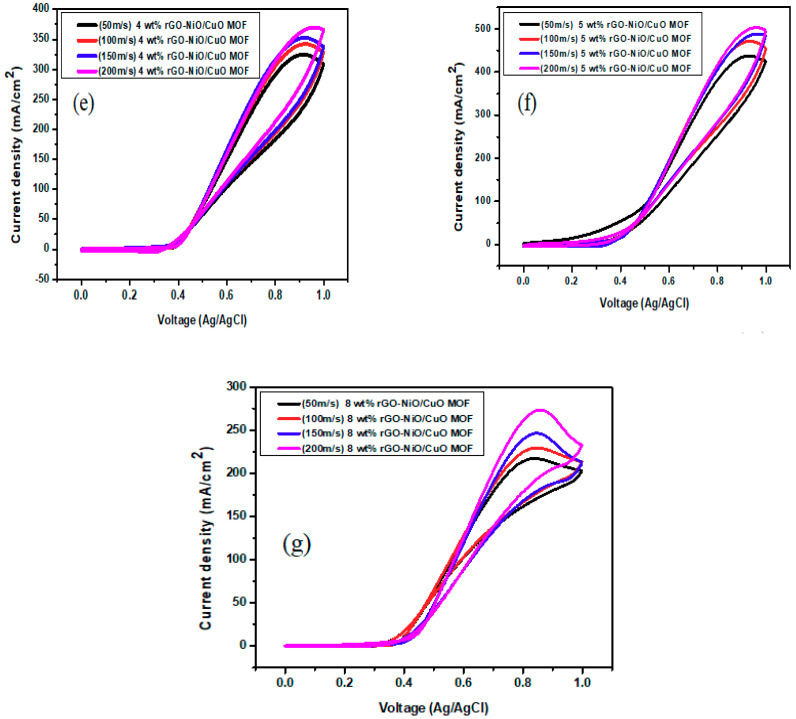
Cyclic Voltamograms of (**a**) NiO/CuO MOF and its composites with (**b**) 1 wt% (**c**) 2 wt% (**d**) 3 wt% (**e**) 4 wt% (**f**) 5 wt% (**g**) 8 wt% rGO-NiO/CuO MOF at scan rate 50–200 mV/s in 3M methanol and 1M NaOH.

Figure 6 illustrates a linear relationship of current density of NiO/CuO MOF and their composite 1–5, 8 wt% rGO–NiO/CuO MOF with increasing scan rate values i.e., 50 mV/s, 100 mV/s, 150 mV/s, 200 mV/s. It was observed that both the peak current density and peak potential were altered by scan rates. With the increase in scan rate the peak current density increases indicating progressive improvement in the electro active specie access towards the electrode surface. Meanwhile the peak potentials shift towards lower negative values, indicating the structural change in the electrochemically formed surface film and saturation of active sites on modified GCE surface was a result of the shifting of peak potential to a more positive value [53]. At higher scan rates, non electro active species were not undergoing oxidation or reduction which resulted in increases in peak current density, suggesting that only electro-active products were formed [54]. In addition, an increase in the scan rate probably improved the electron movement [55]. By comparing the results with gradual increase in scan rate, it was observed that for NiO/CuO MOF, along with all composites, the current density increased accordingly from 50 to 200 mV/s as per expectations. Movement of electrons enhanced by the increasing the scan rate [55] *provide another supportive item of evidence for the increase in current density*. The increases in peak potential shift and peak current density were more prominent in the *5 wt% rGO/NiO/CuO MOF* catalysts compared to the *NiO/CuO MOF and other rGO–based composites i.e., 1–4 wt%, 8 wt% rGO/NiO/CuO MOF* catalyst, representing better efficiency of the catalysts synthesized by the hydrothermal method.

The summary of electrochemical performance of NiO/CuO MOF and its composites with different wt% of rGO and some corresponding values of reported electro catalysts for methanol oxidation reaction are added in Table 2, which represents high activity of NiO/CuO catalyst as compared to previously reported catalysts for methanol oxidation due to conductive characteristics of incorporated rGO.

**Figure 7:**
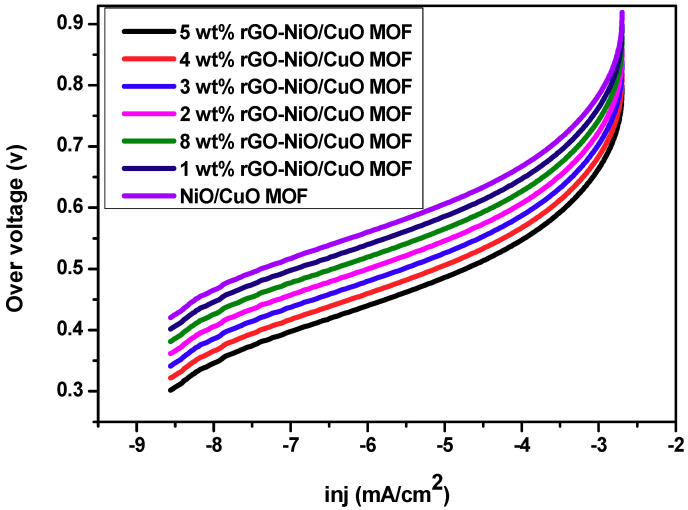
Tafel plots of NiO/CuO MOF and its composites 1 wt%, 2 wt%, 3 wt%, 4 wt%, 5 wt% and 8 wt% rGO in 3M methanol and 1M NaOH at 50 mV/s.

Reaction kinetics of the catalytic process can be determined by Tafel plots, by comparing over potential with current density as shown in Figure 7. It is an appropriate method for the evaluation of methanol oxidation reaction (MOR) activity. Over potential is calculated by using the formula (E–E_o_) [58]. Among all the composites (i.e., 1–5 wt% and 8 wt% rGO–NiO/CuO MOF and NiO/CuO MOF), 5 wt% rGO–NiO/CuO MOF shows the lowest over potential value; this suggests that the inclusion of rGO may not only increase the catalyst surface area but it may also make the accessibility of reactant toward electrode easier and consequently, it apprehends more ions and exhibits higher activity for the oxidation of methanol [59,60]. However, the over potential value of 8 wt% rGO–NiO/CuO MOF is low from 1 wt% and NiO/CuO MOF and high from 2, 3, 4, 5 wt%; these results illustrate a good correspondence with the CV results as they show low current density because of agglomeration of rGO sheets that results in lower surface area and may hinder the ions from reaching the electrode surface.

Furthermore, reaction kinetics of catalytic processes were studied by calculating the Tafel slopes at potential (0.45 V) (Table 3). Tafel slopes for NiO/CuO MOF and their composites i.e., (1–5, 8 wt% rGO–NiO/CuO–MOF) are in the range of 45–65 mV/dec at 0.45 V. The slopes values at lower potential may suggest the breaking of first C–H bond in methanol and first electron transfer that describes the rate-determining step [61,62].

**Figure 8:**
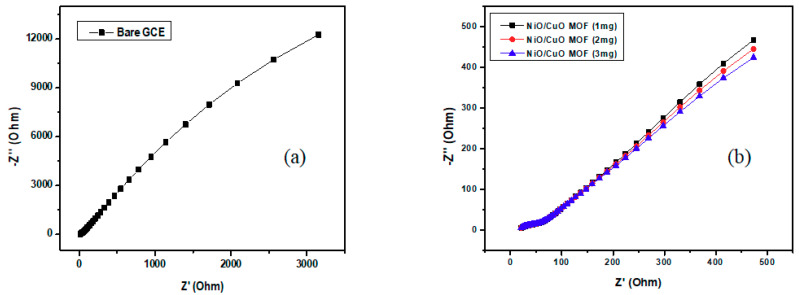

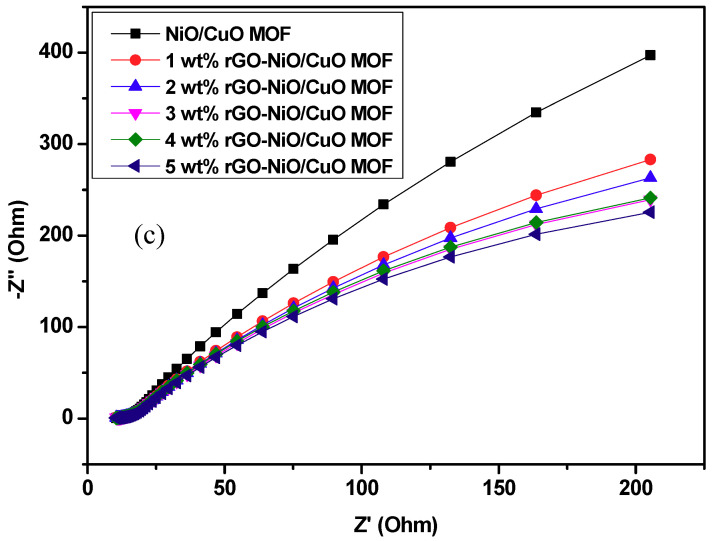
Nyquist plots of (**a**) bare GCE (**b**) NiO/CuO MOF at different concentrations (**c**) NiO/CuO MOF and its composites with 1 wt%, 2 wt%, 3 wt%, 4 wt%, and 5 wt% rGO in 3 M methanol and 1 M NaOH at E = 0.5 V.

Electrochemical impedance spectroscopy (EIS) is a significant technique to demonstrate the electrical conductance of a catalyst by measuring impedance in potentiostatic mode for modified GCE (NiO/CuO MOF and their composites *1, 2, 3, 4, 5 wt% rGO)* in 3M Methanol, 1 M NaOH and with the same three-electrode system. Figure 8 illustrates the plot of bare and modified GCE between real and imaginary impedance, the plot of modified GCE shows a curve in lower frequency area whereas with bare GCE it is just linear as shown in Figure 8a

Nyquist plots for different catalyst loadings (1 mg, 2 mg, and 3 mg) are illustrated in Figure 8b which demonstrates impedance values of NiO/CuO MOF. An inverse relationship was observed between charge transfer resistance and the loading concentrations of catalysts on electrode as charge transfer resistance (R_ct_) decreases with an increase in concentration loading on electrodes which demonstrates the smooth transfer of charges during the electro catalytic oxidation of methanol.

Furthermore, rGO has a significant impact on impedance as incorporation of rGO into NiO/CuO MOF reduces the charge transfer resistance as shown in Figure 8c. Among all composites and MOF, 5 wt% rGO–NiO/CuO MOF shows the lowest R_ct_ value. Moreover, the entire results showed complete compliance with the results of Cyclic Voltammetry [63].

Table 4 exhibits imperative EIS parameters for bare and modified GCE in comparison to a bare working electrode, all synthesized catalysts i.e., NiO/CuO MOF, along with their composites 1–5 wt% rGO–NiO/CuO MOF show low charge resistance and among all combinations, 5 wt% rGO–NiO/CuO MOF shows the lowest R_ct_ (charge transfer resistance) value.

**Figure 9:**
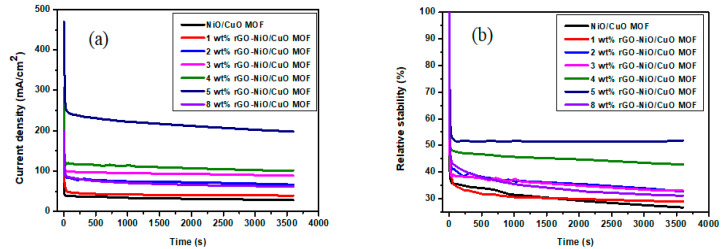
(**a**) Chronoamperometry curves (**b**) relative stability curves of NiO/CuO MOF and its composites with, 1–5 wt% and 8 wt% rGO–NiO/CuO MOF in 3 M methanol and 1 M NaOH at 50 mV/s.

The stability of electro catalysts was observed through chronoamperometry by using the same three-electrode system at a constant voltage of 0.9 V in 3M methanol and 1M NaOH solution as shown is Figure 9a. In the first 60 s there is an initial drop in current density observed mainly because of intermediate reactions i.e., (Co, CHO, COOH) adsorption on catalysts’ surface, after which the current density decreases steadily and reaches a quasi-stationary state until 3600 s. Catalytic sites were vacant at the start of the reaction and were freely available for coverage of methanol but with the passage of time an equilibrium layer of methanol developed on the surface which slowed down the process [64,65]. After applying the set potential for 3600 s, the maximum current density value is (469.4 mA/cm^2^) for 5 wt% rGO-NiO/CuO MOF, followed by 4 wt% rGO–NiO/CuO MOF (306.2 mA/cm^2^), 3 wt% rGO–NiO/CuO MOF (207.2 mA/cm^2^), 2 wt% rGO–NiO/CuO MOF (203.2 mA/cm^2^), 1 wt% rGO–NiO/CuO MOF (136 mA/cm^2^) and NiO/CuO MOF (106.5 mA/cm^2^). While stability of 8 wt% rGO–NiO/CuO MOF was also investigated, this shows a current density value (199 mA/cm^2^) which is comparable with its CV results.

Figure 9b shows % stability with reference to other samples. The catalyst 5 wt% rGO–NiO/CuO MOF which shows highest current density shows highest stability among all the catalysts up to 52% during the time period of 3600 s followed by 4 wt% rGO–NiO/CuO MOF (47%), 3 wt% rGO–NiO/CuO MOF (43%), 2 wt% rGO–NiO/CuO MOF (33%), 1 wt% rGO–NiO/CuO MOF (29%), NiO/CuO MOF (27%) and 8 wt% rGO–NiO/CuO MOF (31%). The above mentioned performance of all samples clearly indicates that addition of rGO in NiO/CuO MOF to a certain limit will assist with the methanol oxidation reaction.

The superior electrocatalytic activity of prepared material for methanol oxidation is based on the synergic effect of the NiO/CuO MOF and rGO sheets. It is reported that methanol oxidation overall process undergoes subsequent necessary elementary steps like methanol adsorption, methanol dehydrogenation into intermediates, and oxidation of these intermediates eventually into carbon dioxide. The process of adsorption occurs in the voids of NiO/CuO MOF, which is further improved by the presence of rGO. For the ease of methanol adsorption and other successive elementary steps for methanol oxidation, surface area of rGO sheets and metal–organic framework porosity aids the process well. Furthermore, adsorption of water molecules in pores of MOF also facilitates the oxidation of intermediates into CO_2_ [66,67].

**Figure 10:**
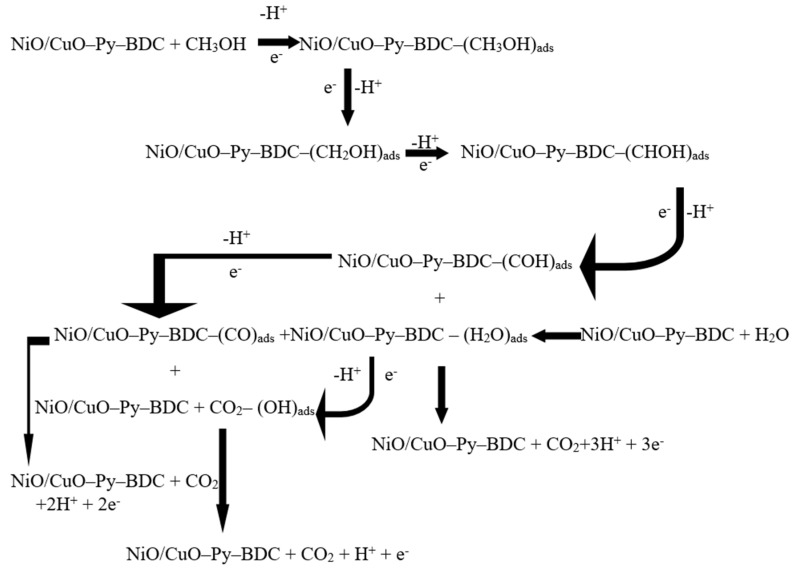
Overall mechanism of methanol oxidation over the surface of catalyst.

A summarized mechanism showing the overall procedure of electrochemical reactions for methanol oxidation on the catalyst surface in 3 M methanol and 1 M NaOH is provided below in Figure 10.

## 6. Conclusions

In summary, this work demonstrated a successful in situ synthesis of novel bimetallic NiO/CuO MOF and its composites i.e., 1–5, 8 wt% rGO–NiO/CuO MOF by economic one-pot hydrothermal method. The confirmation for the coordination of NiO and CuO with BDC and pyrazine ligands was achieved by structural characterizations like FTIR, PXRD, SEM and EDX. Among all synthesized series of catalysts, 5 wt% rGO–NiO/CuO MOF showed the best electrochemical activity with the highest current density of 437.28 mA/cm^2^ at 0.9 V potential, exhibiting low impedance and stability up to 52%. rGO–NiO/CuO–MOF (8 wt%) was also synthesized to optimize the rGO concentration, the catalyst exhibits inferior catalytic activity as compared to 5 wt% rGO–NiO/CuO–MOF due to the higher amount of rGO which resulted in agglomeration. These properties make this bimetallic NiO/CuO MOF a promising alternative to the expensive catalysts for DMFC.

## Figures and Tables

**Table 1:** EDX: Elemental composition of NiO/CuO MOF, 1 wt% rGO–NiO/CuO MOF, 2 wt% rGO–NiO/CuO MOF, 3 wt% rGO–NiO/CuO MOF, 4 wt% rGO–NiO/CuO MOF, 5 wt% rGO–NiO/CuO MOF and 8 wt% rGO–NiO/CuO MOF.

Elements	NiO/CuO MOF	1 wt% rGO–NiO/CuO MOF	2 wt% rGO–NiO/CuO MOF	3 wt% rGO–NiO/CuO MOF	4 wt% rGO–NiO/CuO MOF	5 wt% rGO–NiO/CuO MOF	8 wt% rGO–NiO/CuO MOF
C wt%	29.31	39.29	44.26	47.63	55.70	60.87	67.11
O wt%	18.69	17.79	14.93	10.97	10.57	10.28	10.37
N wt%	8.35	6.42	8.31	7.81	6.63	5.50	4.01
Ni wt%	25.95	22.19	8.24	21.27	14.92	13.02	12.38
Cu wt%	17.70	14.31	5.26	19.13	12.18	10.33	6.13

**Table 2:** Comparison of methanol concentrations, scan rates and current densities along with reported electro catalysts for methanol oxidation.

Catalysts	Methanol Concentration (M)	Loaded Amount of Catalyst (mg)	Scan Rate (mV/sec)	Peak Current (mA/cm^2^)	Reference
ZnO_(40%)_/CeO_2(60%)_dots@CNFs	3	2	50	16.3	[56]
5 wt%GO/Co–MOF-71	3	2	50	29.1	[50]
5 wt% GO/Cu–MOF	3	2	50	120	[57]
5 wt% rGO/NiO–MOF	3	2	50	275.85	[36]
NiO/CuO MOF	3	2	50	67.48	This work
1 wt% rGO–NiO/CuO MOF	3	2	50	145.5	This work
2 wt% rGO–NiO/CuO MOF	3	2	50	259.0	This work
3 wt% rGO–NiO/CuO MOF	3	2	50	264.57	This work
4 wt% rGO–NiO/CuO MOF	3	2	50	324	This work
5 wt% rGO–NiO/CuO MOF	3	2	50	437.28	This work

**Table 3:** Tafel slopes of NiO/CuO MOF and its composites with 1, 2, 3, 4, 5, and 8 wt% rGO–NiO/CuO MOF at 0.45 V.

Catalyst	Tafel Slopes at 0.45 V
NiO/CuO MOF	45.7
1 wt% rGO–NiO/CuO MOF	48.1
2 wt% rGO–NiO/CuO MOF	52
3 wt% rGO–NiO/CuO MOF	55
4 wt% rGO–NiO/CuO MOF	59
5 wt% rGO–NiO/CuO MOF	65
8 wt% rGO–NiO/CuO MOF	50

**Table 4:** Electrochemical parameters extracted from EIS data using bare modified electrodes in 3 M methanol and 1 M NaOH.

Catalyst	Rct (Ohm)	Ru (Ohm)	Cf (F)
Bare GCE	14.06 × 10^3^	18.68	13.37 × 10^−6^
NiO/CuO MOF	634.6	12.77	1.221 × 10^−3^
1 wt% rGO–NiO/CuO MOF	416.6	13.16	1.515 × 10^−3^
2 wt% rGO–NiO/CuO MOF	379.4	13.03	1.547 × 10^−3^
3 wt% rGO–NiO/CuO MOF	337.6	12.84	1.511 × 10^−3^
4 wt% rGO–NiO/CuO MOF	341.7	12.99	1.517 × 10^−3^
5 wt% rGO–NiO/CuO MOF	317.4	12.97	1.597 × 10^−3^

## Supplementary Materials

The following are available online at https://www.mdpi.com/2079-4991/10/8/1601/s1, Figure S1 TGA analysis of pure NiO/CuO MOF.
