# Supplementary material for: Nanocomposites of NiO/CuO Based MOF with rGO: An Efficient and Robust Electrocatalyst for Methanol Oxidation Reaction in DMFC
Source: Nanomaterials (Basel). 2020 Aug 15;10(8):1601. doi: 10.3390/nano10081601 (PMC7466713; doi:10.3390/nano10081601)
Supplement: Supplementary file 1 [file nanomaterials-10-01601-s001.pdf]

Supplementary Information

Nanocomposites of NiO/CuO Based MOF with rGO: An Efficient and Robust Electrocatalyst for Methanol Oxidation Reaction in DMFC

Tayyaba Noor [1,*], Sadaf Pervaiz [2], Naseem Iqbal [3], Habib Nasir [2], Neelam Zaman [3], Muhammad Sharif [4] and Erum Pervaiz [1]

1. School of Chemical & Materials Engineering (SCME), National University of Sciences and Technology (NUST), H-12 Campus, Islamabad 44000, Pakistan; erum.pervaiz@scme.nust.edu.pk
2. School of Natural Sciences (SNS), National University of Sciences and Technology (NUST), H-12 Campus, Islamabad 44000, Pakistan; spervaiz.mschem17sns@student.nust.edu.pk (S.P.); habibnasir@sns.nust.edu.pk (H.N.)
3. U.S-Pakistan Center for Advanced Studies in Energy (USPCAS-E), National University of Sciences and Technology (NUST), H-12 Campus, Islamabad 44000, Pakistan; naseem@uspcase.nust.edu.pk (N.I.); nzaman.ese19ces@student.nust.edu.pk (N.Z.)
4. Department of Chemistry, King Fahd University of Petroleum and Minerals, Dhahran 31261, Saudi Arabia; msharif@kfupm.edu.sa

* Correspondence: tayyaba.noor@scme.nust.edu.pk; Tel: +92-51-90855121

Thermal gravimetric analysis (TGA) was performed using DTG-60H to check the thermal stability of NiO/CuO MOF in a nitrogen atmosphere. Figure 1 shows the weight loss of samples as a function of temperature in nitrogen atmosphere. The TGA curve depicts 5% weight loss until 350 °C that can be ascribed to the evaporation of the adsorbed H_2O molecules (roughly 5%) between 50 and 350 °C. The second mass loss is up to 50% in the temperature range of 350–420 °C resulting from the decomposition of organic ligand. After 420 °C, the mass of material was relatively stable. The high thermal stability of NiO/CuO MOF can be attributed to the coordinative linked interpenetrated framework.

TGA results shows that material is stable up to 350 ^{o}C with the 5% loss of adsorbed water molecules.

The TGA analysis of pure NiO/CuO MOF as shown in figure S1;

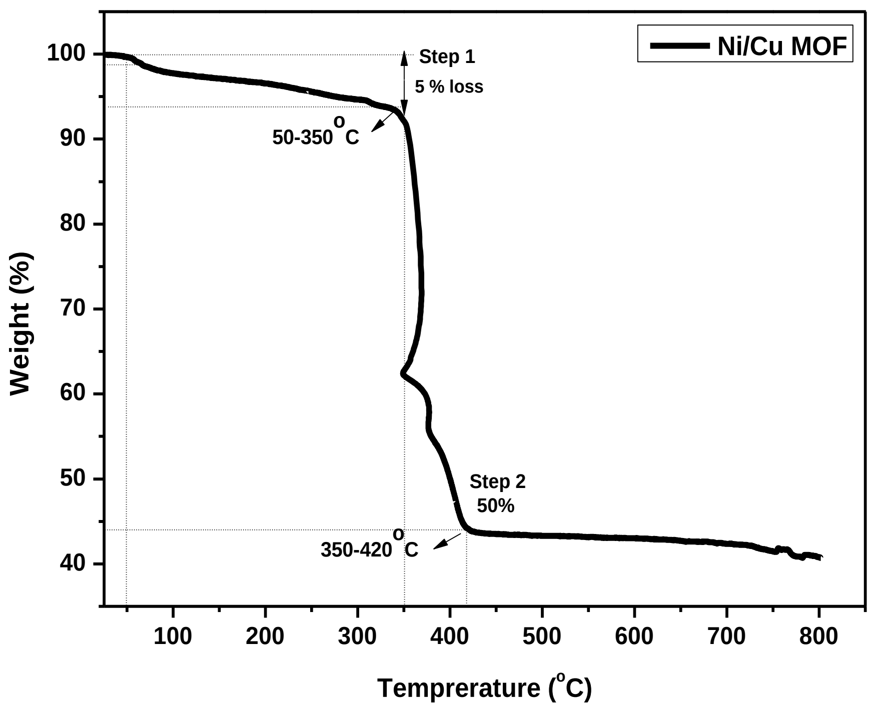

Figure S1. TGA analysis of pure NiO/CuO MOF
